# Supplementary material for: Vitamin D Modified DSS-Induced Colitis in Mice via STING Signaling Pathway
Source: Biology (Basel). 2025 Jun 18;14(6):715. doi: 10.3390/biology14060715 (PMC12190092; doi:10.3390/biology14060715)
Supplement: Supplementary file 1 [file biology-14-00715-s001.zip › Supplementary Materials S1: 16s-report of gut microbiota/Groups/diff_analysis/TaxaMarkers/Order.Groups.sig.boxplot.pdf]

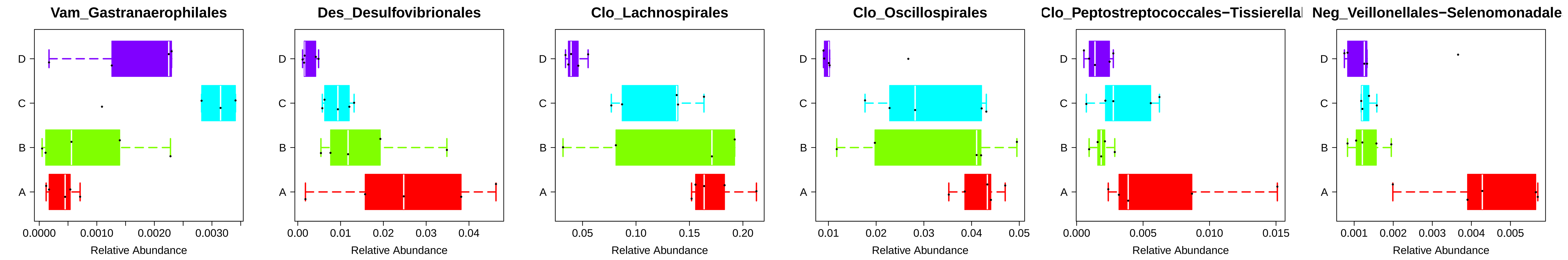
Vam_Gastranaerophilales
Des_Desulfovibrionales
Clo_Lachnospirales
Clo_Oscillospirales
Clo_Peptostreptococcales−Tissierellal
Neg_Veillonellales−Selenomonadale
D
C
B
A
0.0000 0.0010 0.0020 0.0030
0.00 0.01 0.02 0.03 0.04
0.05 0.10 0.15 0.20
0.01 0.02 0.03 0.04 0.05
0.000 0.005 0.010 0.015
0.001 0.002 0.003 0.004 0.005
Relative Abundance
